# Polyaniline modified magnetic nanoparticles coated with dicationic ionic liquid for effective removal of rhodamine B (RB) from aqueous solution†

**DOI:** 10.1039/c8ra06687f

**Published:** 2018-09-26

**Authors:** Mohamad Shariff Shahriman, Nur Nadhirah Mohamad Zain, Sharifah Mohamad, Ninie Suhana Abdul Manan, Suhaila Mohd Yaman, Saliza Asman, Muggundha Raoov

**Affiliations:** Integrative Medicine Cluster, Advanced Medical and Dental Institute (AMDI), Universiti Sains Malaysia 13200 Kepala Batas Pulau Pinang Malaysia; Department of Chemistry, Faculty of Science, University of Malaya 50603 Kuala Lumpur Malaysia muggundha@um.edu.my +603 7967 7022 ext. 2544; University of Malaya Centre for Ionic Liquids (UMCIL), Department of Chemistry, Faculty of Science, University of Malaya 50603 Kuala Lumpur Malaysia; Department of Physics and Chemistry, Faculty of Applied Sciences and Technology, Pagoh Education Hub, Universiti Tun Hussein Onn Malaysia 84000 Pagoh, Muar Johor Malaysia

## Abstract

Polyaniline (PANI) modified magnetic nanoparticle (MNP) nanocomposites coated with newly synthesized dicationic ionic liquid (DICAT) forming MNP-PANI-DICAT were successfully synthesized as a potential material for the removal of Rhodamine B (RB) from water samples. The synthesized material was successfully characterized using a few techniques such as Fourier transform infrared spectroscopy (FT-IR), X-ray powder diffraction (XRD), thermo gravimetric analysis (TGA), Brunauer–Emmett–Teller (BET), and transmission electron microscopy (TEM) analysis. Several parameters have been optimized to enhance the efficiency of the removal process. The adsorption kinetics were investigated and the results showed that MNP-PANI-DICAT was best fitted to a pseudo-second order model for the adsorption of RB. As for the isotherm studies, Temkin's model was found to fit well with the adsorption isotherm of RB on MNP-PANI-DICAT. Other than that, thermodynamics results showed negative values of Δ*G*° for the adsorption of RB, which indicated that the process is thermodynamically feasible, spontaneous and chemically controlled at lower temperature. The negative value of enthalpy Δ*H*° (−40.41) indicated that the adsorption was an exothermic process. The percentage removal of RB was found to be 94.7% by MNP-PANI-DICAT under optimized conditions.

## Abstract 2

Schematic diagram of the removal procedure of RB on MNP-PANI-DICAT.

## Graphical abstract

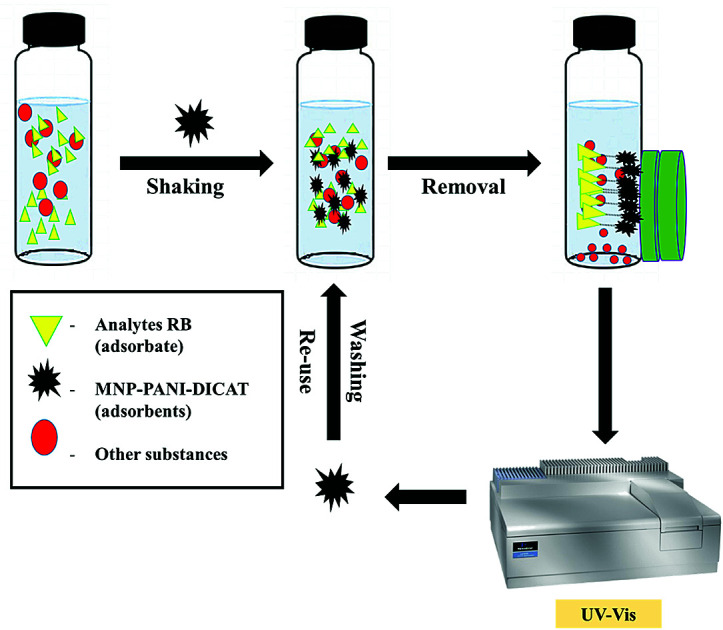

## 1 Introduction

A dye is a coloured, aromatic organic substance which absorbs light in the visible range from 400–800 nm and has been extensively used in the textile and food industries and as a biological stain in biomedical laboratories.^1^ Rhodamine B (RB) is a common hydrophilic organic dye applied for industrial purposes. RB has been forbidden in the food industry for many years due to its suspected carcinogenic nature. RB threatens human health with permanent injury to the eyes, irritation to the gastrointestinal tract with symptoms such as nausea, vomiting and diarrhea and causes methemoglobinemia, cyanosis, convulsions and skin irritation.^2,3^ In addition, because of the expansion of industry and illegal discharges, it still has the possibility of entering the food chain. Therefore, it is significant in environmental science to investigate the removal of RB from water bodies.

Most of the researchers have used various techniques for the removal of dyes from water surfaces. Among all the available method, adsorption techniques are found to be the most promising method for the removal of dyes from water surfaces compared to others.^4^ So far, various effective and efficient adsorbents have been developed to be applied in adsorption studies as an adsorbent material to remove various types of dye compounds.^5,6^ However, most adsorbent materials are difficult to re-collect back from water sample solution. Thus, in this work, RB was removed from water sample using newly synthesized magnetic nanoparticles.

Magnetite (Fe_3_O_4_) nanoparticles (MNPs) showed outstanding performance in term of superior superparamagnetic property, good compatibility, less toxicity and greater of physicochemical stability.^7^ MNPs have gain enormous interest in various fields and applications since emerging as an innovative compound due to its own characteristics.^8–11^ The targeted analytes can be separated by using an external magnetic field from aqueous sample during the adsorption process. However, the application of MNPs in adsorption process is challenging because it can be easily oxidized and agglomerate in aqueous solution. Therefore, some modifications on the surfaces of MNPs either by functionalization or coating with other solid support or functional group such as natural, conductive and synthetic polymers is necessary for better adsorbing capability and extraction ability.^12^

In this study polyaniline (PANI) as synthetic polymer has been introduced to adsorbent materials. Furthermore, PANI have extensively unique characteristics of exchanging between a conductor and an insulator under certain experimental conditions. This polymer can provide better removal application due to its good environmental stability, facile synthesis and relatively low costs.

Ionic liquids (ILs) have been used lately among researchers to increase the extraction efficiencies by loading the ILs on the surface of the polymers that gained an interesting impact in research.^13,14^ Moreover, ILs are known as unique solvent which commonly being used in extraction, synthesis and electrochemistry studies. The significant benefits of using ILs as coating materials are to improve the selectivity and sensitivity toward the targeted analytes by increasing the hydrophobicity and π–π interactions between analytes and the sorbent coated.^15–17^

Monocationic ionic liquid has been extensively studied since few decades ago, but dicationic ionic liquids were less reported.^18–21^ In dicationic ionic liquid form, dication is associated with two identical anions, which make it either exist in hydrophilic or hydrophobic state toward aqueous solution. The dicationic ionic liquid can be classified as symmetric (combination of two identical monocations) or asymmetrical (combination of two different monocations) structures. Other than the uniqueness such as high thermal ability, a wide temperature range of liquid state, and biological activities, the ability of functionalization of dicationic ionic liquids give the possibility of designing more structures with respect to cations, anions and the length of chain linking two cations based on imidazolium and pyrrolidinium.^22,23^

Based on our previous study, we have successfully synthesized new material which is MNP-PANI-DICAT that have been applied as magnetic solid phase extraction (MSPE) for PAHs.^24^ However, we would like to extend the potential of this material for the removal of dye compound. To the best of our knowledge, by combining the excellent properties of PANI modified MNP and coated with new type of dicationic ILs acts as new adsorbents for the removal of RB from water samples have not been reported yet. Herein, in this study MNP-PANI-DICAT has been applied for the removal of RB from aqueous solution and the adsorption mechanisms between the adsorbent and adsorbate have been studied by using kinetic, isotherm and thermodynamics models. The developed removal process has been successfully applied to real water samples.

## 2 Experimental

### 2.1 Chemical and reagent

The selected analyte which is Rhodamine B (RB) was purchased from Sigma-Aldrich (Steinheim, Germany). Ferrous chloride tetrahydrate (FeCl_2_·4H_2_O) and ferric chloride hexahydrate (FeCl_3_·6H_2_O) were purchased from R&M Chemicals, Tamil Nadu, India. Aqueous ammonia (25%) was supplied by Merck, Germany. Ultrapure water (18.2 MΩ cm^−1^) was reproduced by a Sartorius Milli-Q system (Aubagne, France) instruments. All the solvents, potassium peroxodisulfate (K_2_S_2_O_8_) and aniline (C_6_H_5_NH_2_) were purchased from Friendemann Schhmidt Chemical, Perth, Western Australia. Other than that, 1-benzylimidazole, 2,5-dichloro-*p*-xylene and bis(trifluromethane)sulfonamide lithium salt were purchased from Sigma-Aldrich, (Steinheim, Germany).

### 2.2 Material preparations

#### 2.2.1 Synthesis of dicationic ionic liquid-NTf_2_ (DICAT-NTf_2_)

The prepared DICAT-Cl underwent a metathesis process to DICAT-NTf_2_*via* the exchange of anions between Cl and NTf_2_ to change the properties from hydrophilic to hydrophobic state in aqueous solutions. This synthesis of DICAT-Cl and DICAT-NTf_2_ was adopted from our previous work.^24^ Based on Table S1 (ESI†), spectrum of DICAT-NTf_2_ shows the absence of peak at 715.64 cm^−1^ (corresponding to the chlorine group) which indicated the completion of metathesis reaction from DICAT-Cl. Peaks were found to be shifted towards the down field upon the metathesis of DICAT-Cl to DICAT-NTf_2_ in ^1^H NMR spectrum (Table S1, ESI†). DICAT-Cl: percentage yield (93%), melting point (60–62 °C), thin-layer chromatography (TLC): *R*_f_ = 0.6. DICAT-NTf_2_: percentage yield (63%), TLC: *R*_f_ = 0.6. FT-IR and ^1^H NMR spectrums of DICAT-Cl and DICAT-NTf_2_ were assigned well and summarized in Fig. S1 and Table S1 (ESI†).

#### 2.2.2 Synthesis native MNPs, MNP-PANI and MNP-PANI-DICAT

**Fig. 1:**
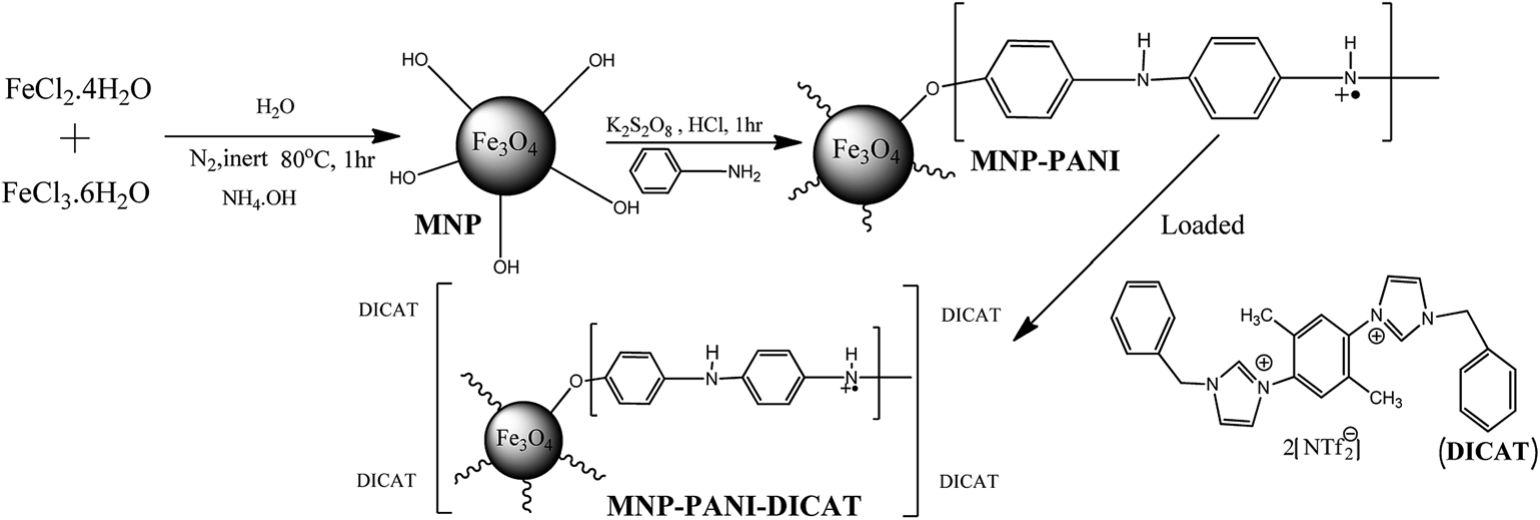
Full proposed schematic representation of MNP-PANI-DICAT.

This synthesis of MNP, MNP-PANI and MNP-PANI-DICAT was adopted from literary works with primary references to Shahriman *et al.* (2018).^24^ The co-precipitation method was selected for synthesized native MNP and self-assembly coating method for MNP-PANI, MNP-PANI-DICAT by coating aniline polymer and dicationic ionic liquids on the surface native MNP. A brief proposed pathways and structure are shown in Fig. 1. The performance of MNP-PANI-DICAT in removal process was compared with native MNP and MNP-PANI toward the removal of rhodamine B.

### 2.3 Instrumentation

The Fourier Transform Infrared (FT-IR) spectra was verified by the Thermo Nicolet FT-IR between 4000 and 400 cm^−1^ using the KBr technique in an absorption mode with 32 scans. Structural interpretation was analyzed with Nuclear Magnetic Resonance (NMR), JEOL 400 MHz (JEOL, Tokyo Japan). The thermal stability was examined by Thermal Gravimetric Analysis (TGA); model TGA-STA 1500, with a heating rate 10 °C cm^−1^ between 25 and 900 °C under nitrogen atmosphere (Perkin Elmer, Massachusetts, USA) The X-ray diffraction (XRD) patterns were verified using an Empyrean X-ray diffractometer using Cu Kα radiation (*λ* = 1.5418*Δ*) at a scan rate of 0.02 s^−1^. The morphological analysis of the synthesized products was executed using Transmission Electron Microscopy (TEM) on FEI CM12 instrument. The Brunauer–Emmett–Teller (BET) analysis was carried out *via* surface area analyzer (Quantachrome, Boynton Beach, FL, USA) to determine specific surface area and pore diameter of nanosorbents. High performance thin layer chromatography (HPTLC, LINOMAT 5, CAMAG, Muttenz, Switzerland) was used to verify the purity of compounds. An Accumet AB150 pH meter (Fisher Scientific) was used for the pH measurements. A Perkin Elmer, Model Lambda 25 Ultraviolet-Visible (UV-Vis) spectrophotometer (Massachusettes, U.S.), equipped with 1 cm quartz cells at 554 nm was used for removal of RB. The parameters set for measurements were wavelength accuracy ± 0.5 nm, bandwidth 1.0 nm, and scan speed 400 nm min^−1^.

### 2.4 Standard and working solutions

The standard stock solution of RB (1000 mg L^−1^) was prepared in acetonitrile (ACN) and stored in a refrigerator to avoid the degradation process. The working standard solutions containing all the analytes were freshly prepared by dilution of the stock solutions with ultrapure water.

### 2.5 Batch experiments

#### 2.5.1 Batch adsorption studies

MNP-PANI-DICAT (20 mg) was placed in storage vial that contained 10 mL of an aqueous solution of analyte at an identified concentration (10 mg L^−1^). The solution was shaken at 250 rpm for 120 min at room temperature to allow the RB to attract toward the binding sites of MNP-PANI-DICAT. Subsequently, external magnetic field was used to separate the MNP-PANI-DICAT from sample solution. The supernatant was collected upon formation of clear solution and the concentration was analysed by using UV-Vis. The percentage of removal, *R* (%), and the amount of analyte adsorbed per unit mass of the adsorbent (*q*_e_) were calculated as follows in eqn (1) and (2):1
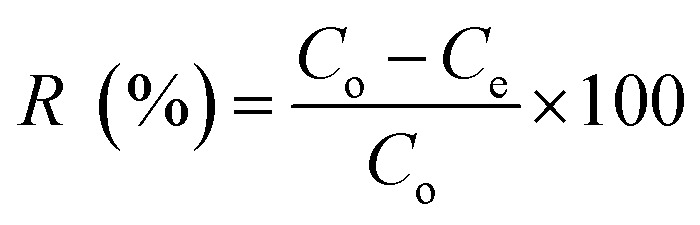
2
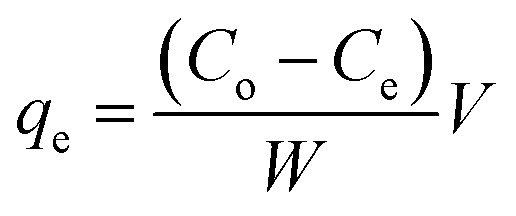
where, *C*_e_ and *C*_o_ are the equilibrium and initial concentration of solutions (mg L^−1^), respectively. The *W* (g) is the mass of the dry adsorbent used and *V* (L) is the volume of the solution.

#### 2.5.2 Optimization of adsorption study

Effect of pH was anticipated in the scope of pH (1–10) at room temperature. The desired pH was balanced with 1 M HCl and 0.1 M NaOH utilizing pH meter. The adsorbent dosage was fixed at 20 mg with initial concentration of RB at 10 mg L^−1^ in 10 mL of aqueous solution. The influence of contact time on the adsorption of the RB has been investigated at various time intervals (0–180 min) at 298 K. The adsorbent dosage was fixed at 20 mg with initial concentration of RB at 10 mg L^−1^ in 10 mL of aqueous solution at pH 1. The equilibrium studies were carried out at various RB initials concentrations (5–100 mg L^−1^) with adsorbent dosage was secure at 20 mg in 10 mL of aqueous solution at pH 1. The experiments were carried out at 298, 318, and 338 K, respectively for thermodynamic study.

### 2.6 Real sample analysis

Environmental water was collected by using schott glass bottles in various places in Penang, Malaysia which are tap water allocation nearby the dye industry, industrial waste water at painting industry and lake water samples nearby with industrial waste water treatment. The reason of collection point for water samples from industrial waste water because industrial painting using dyes extensively. For collection of the lake water due to place nearest to industrial waste water treatments. Moreover, tap water sample was collected to investigate the matrix effect for the removal of RB from aqueous solution. All the water samples were filtered immediately using a nylon 0.45 μm membrane. The desired pH 1 with 60 min of contact time was selected for the real sample analysis. The spiking concentration for real sample analysis was 80 mg L^−1^ at room temperature as equilibrium conditions for MNP-PANI-DICAT. RSD were achieved using three different vials as triplicates (*n* = 3).

### 2.7 Reusability for adsorption study

To examine the probability of recycling and renewing the sorbent (MNP-PANI-DICAT) five cycles of adsorption experiments were carried out using the same adsorbent. Each recycling process started after the material was washed with acetonitrile (ACN) and then dried before each removal process.

## 3 Result and discussions

### 3.1 Characterization of materials

**Fig. 2:**
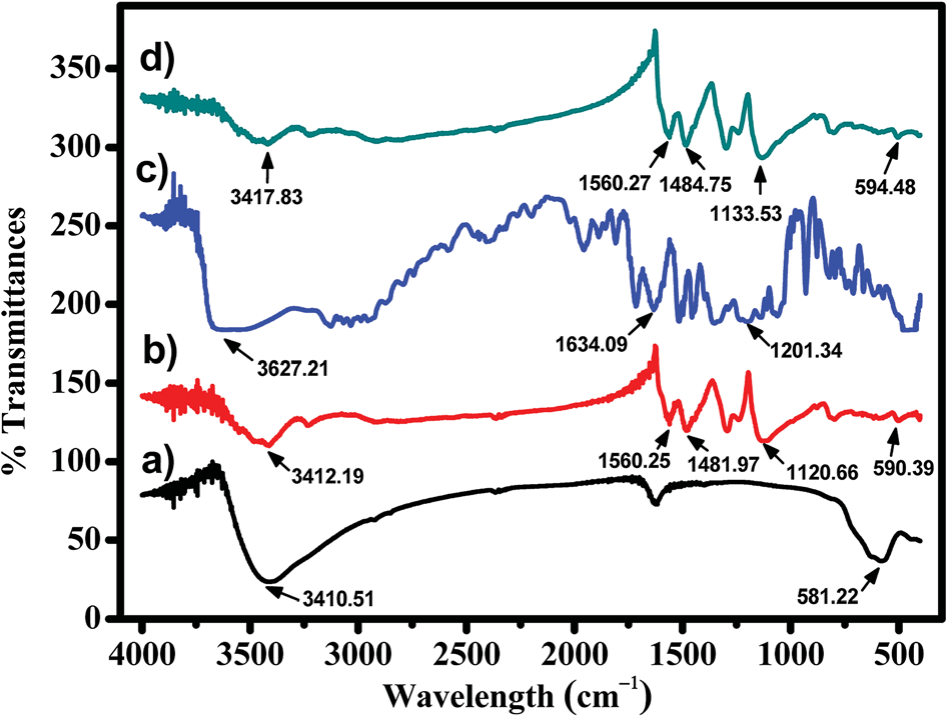
FTIR spectra of (a) MNPs, (b) MNP-PANI, (c) DICAT-NTf_2_ and (d) MNP-PANI-DICAT.

The FT-IR spectra in Fig. 2 show several additional peaks in the spectrum of MNP-PANI-DICAT, proportional to the MNPs and MNP-PANI spectrum, indicating a successful coating process of DICAT on MNP-PANI. Fe–O and O–H stretching vibrations on MNPs spectrum focusing at peaks 581.22 cm^−1^ and 3410.51 cm^−1^, respectively. The presence of C=C aromatic ring of PANI was showed in MNP-PANI (1481.97 cm^−1^) and MNP-PANI-DICAT (1484.25 cm^−1^). Moreover, peak of quinoid (N=C=N) ring of PANI, was observed in MNP-PANI (1293.20 cm^−1^) and MNP-PANI-DICAT (1298.51 cm^−1^), which further proves the coating of PANI on MNP. Upon the loading of DICAT-NTF_2_, the C–N aromatic ring of ionic liquid was clearly observed at 1133.53 cm^−1^ in the FT-IR spectrum of MNP-PANI-DICAT. The strong N–H bond of MNP-PANI and MNP-PANIDICAT on aromatic ring at 1560 cm^−1^ showed that aniline was in the polymer network structure. Hence, the FT-IR analysis clearly proved that the formation of MNP-PANI-DICAT was successful.

Microscopic morphological structures were performed by using TEM technique to determine and compare the surface features of MNP, MNP-PANI and MNP-PANI-DICAT. Based on TEM analysis as shown in Fig. S2 (ESI†), average diameter of the particles as found to be in the increasing in the order of MNP (4.6 nm), MNP-PANI (5.0 nm) and MNP-PANI-DICAT (6.8 nm). Furthermore, ionic liquid itself increased the particles size, which can give more binding sites on the MNP-PANI-DICAT due to dicationic IL which have larger cation on it that can increase the size particles of the materials.^25^ This phenomenon could be related in BET analysis that MNP-PANI-DICAT have heterogeneous surface with aniline polymer and imidazolium on top of magnetic core–shell.

**Fig. 3:**
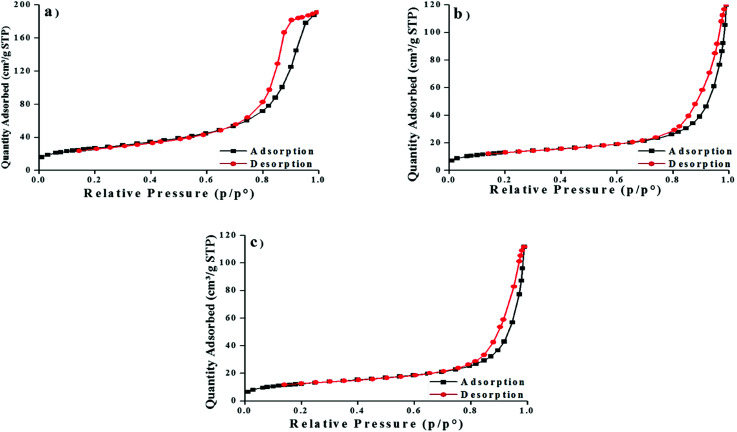
Nitrogen adsorption–desorption isotherms of (a) MNP, (b) MNP-PANI and (c) MNP-PANI-DICAT.

The BET surfaces area showed high volume of adsorption on MNP than MNP-PANI and MNP-PANI-DICAT which indicated MNP (96.9882 m^2^ g^−1^) has larger surface area compared to MNP-PANI (46.9048 m^2^ g^−1^) and MNP-PANI-DICAT (45.1510 m^2^ g^−1^), respectively as shown in Fig. 3. This finding could be related with the covering of the adsorption sites by organic moieties which have immobilized on the surface of MNPs and further hindered the N_2_ molecules from accessing to the binding site.^14^ Meanwhile, pore diameters which calculated from Barret–Joyner–Halenda model (BJH) were found to be increasing from MNP, MNP-PANI and MNP-PANI-DICAT which existed in the mesoporous region. Mesoporous materials (between 2 and 50 nm) which have pore size between microporous (smaller than 2 nm) and macroporous (larger than 50 nm) materials. Mesoporous materials itself have more advantages such high surface area, a lot of pore volume on materials and have good stability compared to microporous and macroporous materials.

**Fig. 4:**
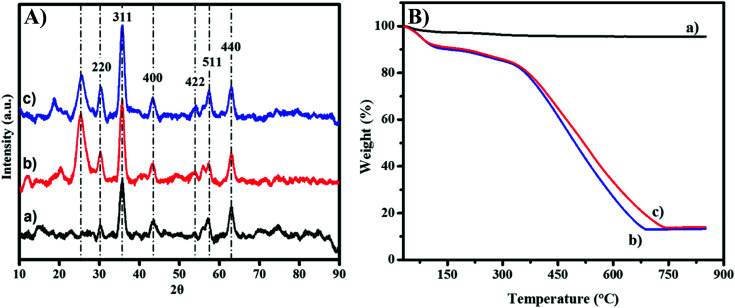
(A) XRD and (B) TGA analysis of materials, (a) MNPs, (b) MNP-PANI and (c) MNP-PANI-DICAT.

The crystallinity analysis was conducted by using X-ray powder diffraction (XRD) as plotted in Fig. 4A. The diffraction peaks of MNP-PANI and MNP-PANI-DICAT are sharp and slightly wider than unmodified MNPs, could be due to the present of amorphous and polymeric materials coated on the surfaces of MNPs.^26^ Native MNPs indicated good crystallinity, with diffraction peaks appearing at the 2*θ* values of 30.5°, 36.5°, 43.6°, 54.3°, 57.4° and 63.0° which can be allocated to the (220), (311), (400), (422), (511) and (440) cubic spinel planes of Fe_3_O_4_, respectively according to JCPDS card number 88-0866.^27^ Furthermore, the intensity of diffraction peaks, MNP-PANI and MNP-PANI-DICAT were observed to be increase in crystalline phase which proved that the product is successfully formed.^28^

Meanwhile, MNP, MNP-PANI and MNP-PANI-DICAT were undergo analysis by using Thermo-Gravimetric Analysis (TGA) to measure a compound's thermal stability. Fig. 4B and Table S2 (ESI†) shows the weight-loss curves for MNP-PANI and MNP-PANI-DICAT involve a few steps process and for native MNPs only one step process. Generally, the first step can be interpreted due to the loss of water, the second step may account for most weight of the polymer. Native MNPs do not show the significant weight loss until it reaches elevated temperature. Fig. 4B, showed that the second degradation stage of MNP-PANI-DICAT polymer can be sustain at higher temperature (364–739 °C) with low weight loss (66.6%), which further proved that MNP-PANI-DICAT polymer is more stable than MNP-PANI and native MNPs. Furthermore, the higher stability could be due to strong interaction between polyaniline and dicationic ionic liquid on core of MNP, which make it more stable at higher temperature.

### 3.2 Preliminary adsorption studies

**Fig. 5:**
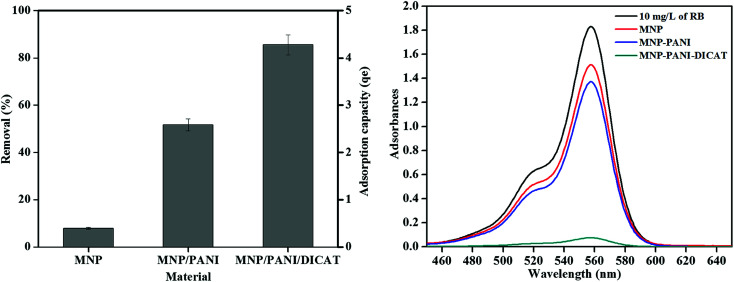
Removal of RB and scanning wavelength in preliminary batch sorption experiments. (Condition: 298 K, 10 mL of 10 mg L^−1^ analytes solution, 20 mg sorbent, and 250 rpm with 120 min shaking times).

The synthesized magnetic nanoparticles have been applied in the adsorption of rhodamine B (RB) and the performance of MNP-PANI-DICAT, MNP-PANI and native MNPs was compared. The percentage of removal of RB by using the synthesized magnetic nanoparticles was presented in Fig. 5. Based on the graph, MNP-PANI showed higher removal percentage and adsorption capacity that might due to aniline polymer that coating around magnetic nanoparticles that allow it increased in term of porosity of the adsorbent to attract and captured more RB particles toward it. It was also found that the MNP-PANI-DICAT exhibits higher percentage of removal (%) and adsorption capacity (*q*_e_) compared to the native MNPs and MNP-PANI due to the presence of dicationic ionic liquid in MNP-PANI-DICAT polymer that might increase the selectivity towards RB. Furthermore, higher percentage of removal was observed due to strong π–π interaction between the aromatic ring of RB and imidazolium ring in MNP-PANI-DICAT.

### 3.3 Effect of pH

**Fig. 6:**
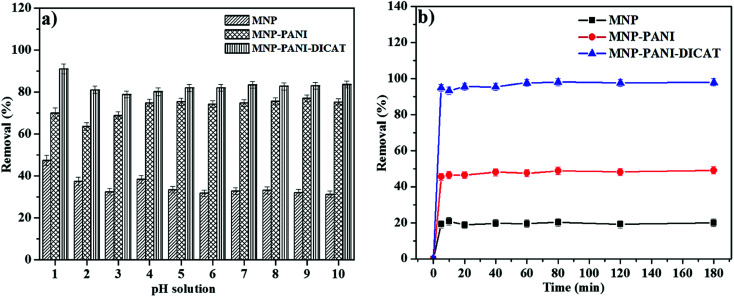
(a) Effect of initial pH (b) effect of initial time on RB. (Condition: sorbent: 20 mg; initial concentration: 10 mg L^−1^; volume: 10 mL; sample pH at pH 1, removal time: 60 min, and temperature 298 K).

One of the extreme crucial features in adsorption investigations, which has been described by many researchers, is the substantial role of pH in adsorption efficiency. The effect of pH on the adsorption of RB using MNP-PANI-DICAT was studied by varying the pH of RB solution from pH 1 to pH 10. Based on results in Fig. 6a, maximum percentage removal of RB was obtained in an aqueous solution at lowest pH (pH 1) probably because lower pH created more H^+^ ions surrounded the surface of the MNP-PANI-DICAT.^29,30^ In term of sorption state properties, the influence of pH toward RB solution on the surface of MNP-PANI-DICAT at lower pH ranges leads to chemisorption state along with physisorption state.^31^

Based observation, the percentage removal of RB in an aqueous solution was slightly reduced while, increased of pH until pH 3 and not significantly increased from pH 4 to 10.^32^ This is because the presence of OH^−^ groups changed the form of RB structure. At this condition, the zwitterionic form of RB in the solution were formed that might change the properties of RB which may lead to aggregation into larger molecules form (dimer) of RB in solution.^32^ Thus, this created difficulty for the dimer RB to enter the pore structure on surface of MNP-PANI-DICAT. Moreover, chemisorption along with physisorption state of RB tend to occur at higher pH.^31^ Some comparable results have been testified in the literature.^33–35^

The maximum sorption capacity of RB by MNP-PANI-DICAT at pH 1 make it as an excellent sorbent compared to MNP-PANI and native MNPs.

### 3.4 Effect of contact times

The time ranges effect was examined from 5 to 180 min for the removal of RB using MNP-PANI-DICAT. As shown in Fig. 6b, it detained RB was increase at first 5 min until 60 min since more adsorption sites available at MNP-PANI-DICAT and the percentage of removal went up to 95%. The MNP-PANI and native MNPs shows increment until it reaches equilibrium point at 60 min with 47.6% and 20% of removal, respectively. By further increment from 80 to 180 min, no significant changes were observed in percentage of removal that could be due to saturated binding sites on surface of MNP-PANI-DICAT and same trend was obtained for MNP-PANI as well. As for MNP, 20% removal of RB was observed up to 180 min due to less of number pores and this will reduce the adsorption sites that available on surface of MNP compared to MNP-PANI and MNP-PANI-DICAT.

Effect of contact time was also presented by referring to UV-Vis spectrum for the removal of RB by MNP, MNP-PANI and MNP-PANI-DICAT in Fig. S3 (ESI†). The absorbance values were found to be decrease as the adsorption time increase, and this can be obviously seen for MNP-PANI-DICAT. In this work, the percentage removal of RB was found to obtained equilibrium at 60 min for all the studied adsorbents. Therefore, 60 min was selected as an optimum time for this adsorption process.

### 3.5 Adsorption kinetic models

The kinetic and mechanism parameters were investigated for the adsorption of RB onto native MNPs, MNP-PANI and MNP-PANI-DICAT. Five several types of models were carried out which are pseudo first order,^9,36,37^ pseudo second order,^38^ Elovich,^39^ intra particles and external diffusion model.^40^ The normalized standard deviation value, Δ*q* (%) which determined from eqn (3) and relative error (%) determined from eqn (4) were acquired to define the fitness of the model to designate the adsorption kinetics, which definite as:3
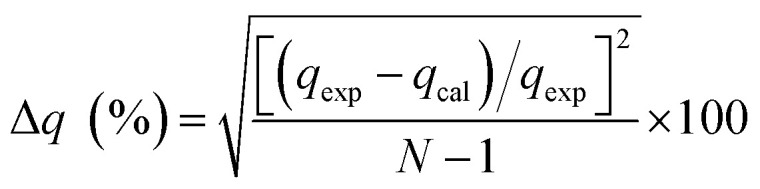
4
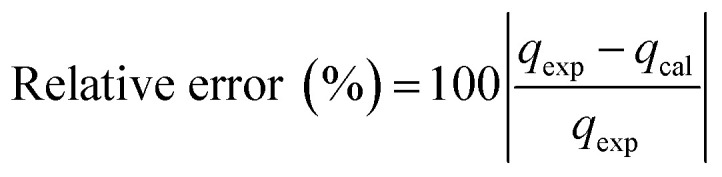
where *N* signifies the number of data points, while *q*_exp_ and *q*_cal_ (mg g^−1^) are the experimental and calculated adsorption capacities, correspondingly. Lower the value of relative error (%) and Δ*q*, the better the model fits.^39,41^

#### 3.5.1 Pseudo first order model

Pseudo first order was developed and extensively applied in adsorption of numerous solutes from aqueous solution onto different solid adsorbents.^42^ This kinetic equation of pseudo first order that described the adsorption of liquid–solid systems founded on the solid capacity.5
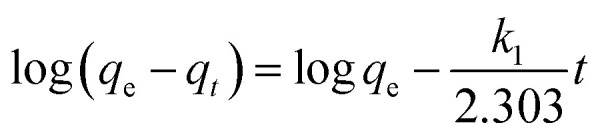
where *q*_e_ (mg g^−1^) is the amount of equilibrium uptake, *q*_*t*_ (mg g^−1^) is the amount of solute adsorbed at any time *t*, *k*_1_ is the rate constant of pseudo first order model (min^−1^) and is specified in eqn (5). If the experimental data can be fitted well with pseudo first order, graph plotted with log(*q*_e_ − *q*_*t*_) *versus t* is a straight line. Generally, when adsorption is initially introduced by diffusion through a boundary, the kinetics will follow pseudo first order model in most cases.^43^6
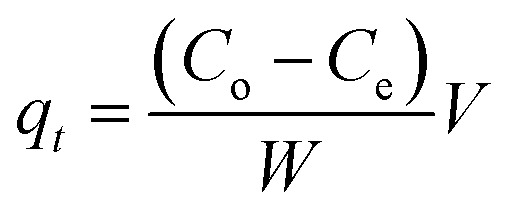
where, *C*_o_ and *C*_e_ are the initial and equilibrium concentration of solutions (mg L^−1^), separately. *W* (g) is the mass of the dry adsorbent used and *V* (L) is the volume of the solution for eqn (6).

#### 3.5.2 Pseudo second order model

This model equation based on equilibrium adsorption can be listed as eqn (7):^38^7
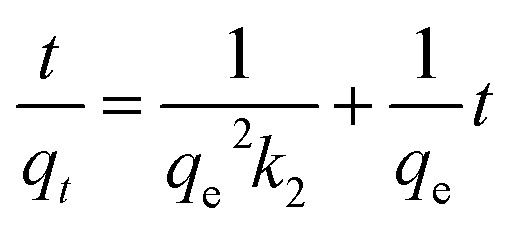


**Fig. 7:**
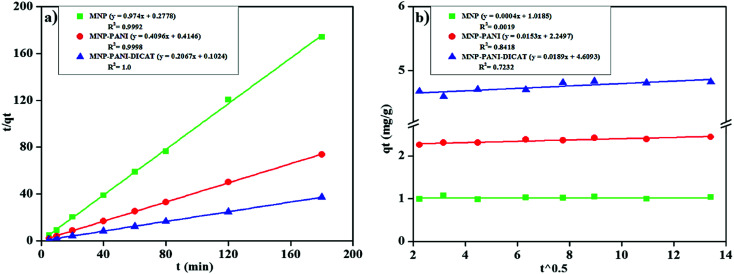
(a) Pseudo second order model (b) intraparticle diffusion model for the adsorption of RB on native MNP, MNP-PANI and MNP-PANI-DICAT at 298 K.

The plot of 
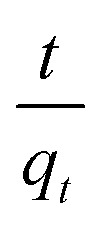
*versus t* gives a straight line with 
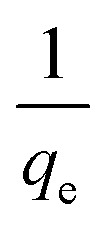
 and 
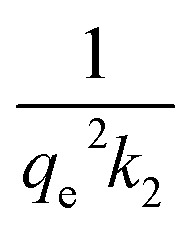
 as the slope and intercept if pseudo second order is applicable. *k*_2_ and *q*_e_ can be obtained directly from the graph without having to know the parameters previously. This kinetic model is more likely to apply onto the whole range of adsorption studies and it shows that chemisorption is the rate determining step.^38,43^ The kinetic parameters of different models are shown in Table 1 with correlation of determination (*R*^2^), normalized standard deviation, Δ*q* (%) and relative error (%) for the adsorption of RB. Based on Table 1, the well fitted model is pseudo second order kinetics model with lower Δ*q* value and relative error (MNPs = 1.8580% and 4.9158%, MNP-PANI = 0.0023% and 0.0061%, MNP-PANI-DICAT = 0.2066% and 0.5467%), respectively. Moreover, the calculated *q*_e_ values were closely fitted the experimental data. The correlation of determination (*R*^2^) values ranging from 0.9992–1.0000, which further supported that the adsorption of RB on the MNP-PANI-DICAT followed the pseudo second order model (Fig. 7a). In comparison, pseudo first order model shows the enormous difference between the experimental and the calculated adsorption capacity for RB that represented by Δ*q* (%) as shown in Table 1.

**Table 1:** Details of kinetic parameter and coefficient of determination for various kinetic models for the adsorption of RB onto MNP-PANI-DICAT

Kinetic models	Parameters	Materials		
		MNP	MNP-PANI	MNP-PANI-DICAT
	*q*_e,exp_ (mg g^−1^)	1.0798	2.4416	4.8116
Pseudo-first-order	*q*_e,cal_ (mg g^−1^)	0.1532	0.0720	0.1622
	*k*_1_ (min^−1^)	0.0085	−0.0058	−0.0023
	*R* ^2^	0.2415	0.0867	0.0058
	Δ*q* (%)	32.4340	36.6820	36.5220
	Relative error (%)	85.8120	97.0530	96.6290
Pseudo-second-order	*q*_e_, cal (mg g^−1^)	1.0267	2.4414	4.8379
	*k*_2_ (g mg^−1^ min^−1^)	3.4149	0.4047	0.4172
	*h* (mg g^−1^ min^−1^)	3.5597	2.4119	9.7656
	*t* ^1/2^ (min)	3.3261	0.1658	0.0862
	***R*** ^**2**^	**0.9992**	**0.9998**	**1.0000**
	**Δ*q* (%)**	**1.8580**	**0.0023**	**0.2066**
	**Relative error (%)**	**4.9158**	**0.0061**	**0.5466**
Elovich equation	*q*_e_ cal (mg g^−1^)	1.0215	2.3818	4.7726
	*β* (g mg^−1^)	909.0910	20.2840	16.6945
	*α* (mg g^−1^ min^−1^)	—	7.8713 × 10^17^	3.9980 × 10^31^
	*R* ^2^	0.0190	0.9016	0.7433
	Δ*q* (%)	2.0400	0.9242	0.3064
	Relative error (%)	5.3974	2.4452	0.8105
Intra-particle diffusion	*C* (mg g^−1^)	1.0185	2.2497	4.6093
	*K* (mg g^−1^ min^−1^)	0.0004	0.0153	0.0189
	*R* ^2^	0.0019	0.8418	0.7232
External diffusion	*k*_ext_ (1/min)	7.0 × 10^−6^	0.0003	0.0064
	*C* (mg g^−1^)	−0.2185	−0.6225	−2.9960
	*R* ^2^	0.0026	0.7219	0.6448

#### 3.5.3 Elovich's model

To further prove in term of chemisorption, the Elovich's model was applied. The Elovich model calculation is as displayed below eqn (8):^39^8
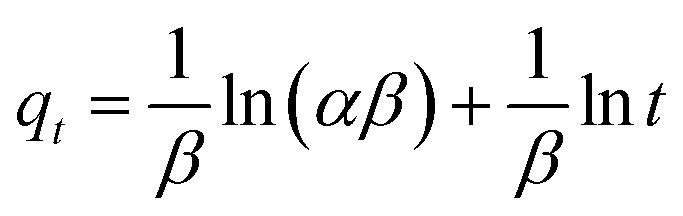
where the underlying sorption amount (mg g^−1^ min^−1^) represent by *α*, *β* is identified with the expanded exterior exposure, and performing vitality for chemisorption (g mg^−1^). Through linear plot *q*_*t*_*versus* ln *t*, estimation of *α* and *β* can be acquired. The *R*^2^ which more than 0.74, for RB adsorption on MNP-PANI-DICAT with the calculated adsorption capacity and closeness of experimental also supported toward pseudo second order model.

#### 3.5.4 Intra particle diffusion

Internal or within particle diffusion has represented as rate of diffusion at early stage on adsorption in Fig. 7b. A straight-line plot of *q*_*t*_*versus t*^0.5^ that passes through origin was indicate the rate-controlling step. The Weber and Morris' model condition can be composed as eqn (9):^40^9
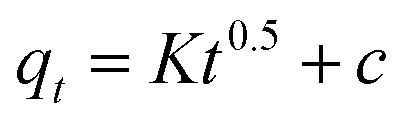
where, *K* is the intra particle diffusion rate constant (mg g^−1^ min^−1^) and *c* represent the intercept (mg g^−1^). The results showed that no straight line passing through the origin for adsorption of RB on native MNPs, MNP-PANI and MNP-PANI-DICAT, assumed that intra particle diffusion was not involved at rate of controlling step but in sorption process only. Besides that, the value of *c* for MNP-PANI-DICAT (4.6093 mg g^−1^) is higher revealed that superior adsorption mechanism happened toward RB solution compared to MNP-PANI and native MNPs.

#### 3.5.5 External diffusion

The external or film diffusion model can be evaluated when zero intercept at origin in the graph of ln (*C*_*t*_/*C*_o_) *versus t*. The equation of external diffusion has described as follows in eqn (10):10
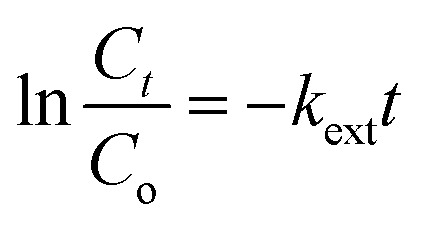
where *C*_*t*_ and *C*_o_ indicate the concentration of the solute in the liquid phase at time, *t* and initial solution and *k*_ext_ (1/min) is a diffusion rate parameter. The external/film diffusion also showed not applicable in the sorption process due to the plotting straight line which did not intercept the origin point (Table 1).

In conclusion, the model was fitted well in the kinetic data for the adsorption of RB, in the order of pseudo second order kinetics > Elovich > pseudo first order kinetics. The intra particle and external diffusion model simultaneously involved in the adsorption process but it is not at rate determining step.

### 3.6 Effect of initial concentration (*C*_o_)

**Fig. 8:**
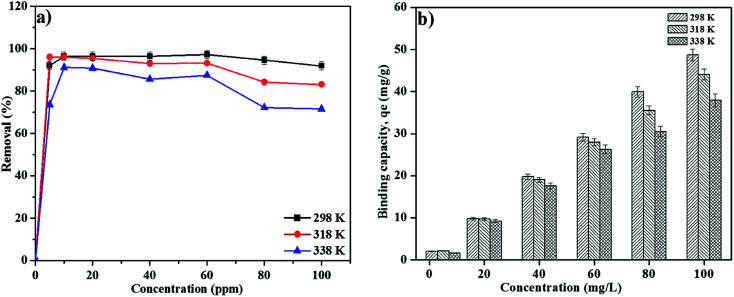
(a) Effect of initial concentration RB (b) effect of solution temperature with different initial concentration on RB on MNP-PANI-DICAT. (Condition: sorbent: 20 mg; volume: 10 mL; sample pH at pH 1, and removal time: 60 min).

The effect of initial concentration of RB on MNP-PANI-DICAT was studied in the series of 5–100 mg L^−1^ at different temperatures, separately (Fig. 8a). The mesoporous on the surface of MNP-PANI-DICAT was favourable to reducing the mass transfer resistance and had become useful for fast RB adsorption. The percentage of removal for RB was found to be increase rapidly up to 10 mg L^−1^ and constant until 40 mg L^−1^. The availability of more active binding sites on the MNP-PANI-DICAT than the number of RB ions in the solution could contributed to the increasing of percentage of removal of RB. The percentage of removal achieved equilibrium at 80 mg L^−1^ and constantly for adsorption of RB was observed with further increase in concentration of RB. This could be due to the saturation of binding sites of MNP-PANI-DICAT toward RB ions in aqueous solution. Therefore, the concentration of 80 mg L^−1^ at room temperature was selected for further experiments.

### 3.7 Effect of solution temperature

As shown in Fig. 8b the binding capacity, *q*_e_ (mg g^−1^) of RB on MNP-PANI-DICAT was found to decline at elevated temperature in all concentrations which inspected from 5 to 100 mg L^−1^. Below 40 mg L^−1^ showed, there are no significant difference between desire temperatures. Fig. 8b indicates below 60 mg L^−1^ concentration of RB, slightly difference on adsorption capacity between three different temperatures was observed, but at higher than 60 mg L^−1^ concentration of RB, adsorption capacity was found to be optimum at 298 K. Furthermore, at higher temperatures, it might reduce the tendency to be adsorbed on binding sites of MNP-PANI-DICAT adsorbent due to agitation or degradation of RB solution. Hence, 298 K was set up to be the equilibrium temperature for the removal of RB using MNP-PANI-DICAT adsorbent.

### 3.8 Adsorption isotherm models

The interaction between adsorbate with an adsorbent was investigated by using adsorption isotherm models. Thus, the results obtained from the equilibrium isotherm studies is essential to evaluate the affinity of adsorbent and adsorbate in aqueous solution. There are few tested modelling was carried out in past which are Langmuir,^44,45^ Freundlich,^46^ Temkin,^47^ Dubinin–Radushkevich's,^48^ and Halsey,^49^ isotherm equations.

The equilibrium of the adsorption of sorbate on the surfaces of an adsorbent at a given pH and temperatures was described by using adsorption isotherm models. Isotherm study can barely describe based on plotting of the compound concentration stability in the adsorbent as a function of its equilibrium concentration solution.^50^*R*^2^ value which is adjoining to unity demonstrates that the isotherm model contributes the perfect fit to the investigational data.

The isotherm adsorption model was used to describe how sorbates interact with the adsorbent by optimizing the use of the adsorbent. In this study, Langmuir, Freundlich, Dubinin–Radushkevich, Temkin and Halsey models have been studied.

#### 3.8.1 Langmuir's model

The contaminations sorption lead to the Langmuir that has been take into consideration. It depends on the suspicion concerning the monolayer adsorptions on a homogenous surface with uniform energies of adsorption for all the binding sites. In addition, no more adsorption process would happen if the site is possessed by a solute.^51^ The Langmuir isotherm equation in linear form can be signified by the following equation in eqn (11):^52^11
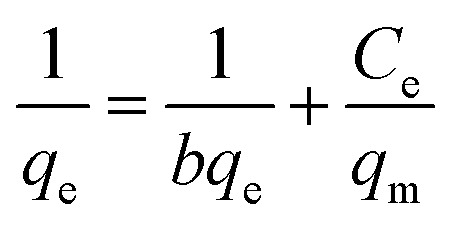
where *C*_e_ (mg L^−1^) is the equilibrium concentration of the adsorbate, *C*_o_ (mg L^−1^) is the underlying adsorbate concentration, *q*_e_ (mg g^−1^) is the adsorption capacity at equilibrium, *q*_m_ (mg g^−1^) and *b* (L mg^−1^) are Langmuir consistent identified with the adsorption limit and the rate of adsorption, separately. To determine if the adsorption process is favorable or unfavorable, dimensionless separation factor (*R*_L_) is calculated using the following equation in eqn (12).^51^ The Langmuir's model parameters were determined and listed in Table 2.12
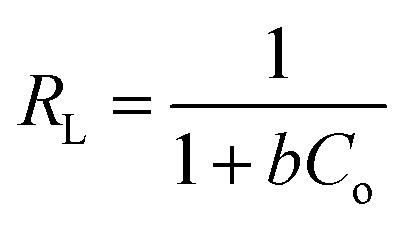


**Table 2:** Details of isotherm constants and correlation coefficient of determination for various adsorption isotherms for the adsorption of RB onto MNP-PANI-DICAT

Isotherm models	Parameters	Temperatures (K)		
		298 K	318 K	338 K
Langmuir	*q*_m_ (mg g^−1^)	109.8901	51.0204	59.5238
	*b* (L mg^−1^)	0.1102	0.2444	0.0558
	*R* ^2^	0.3000	0.9815	0.5169
	*R*_L_	0.1019	0.0487	0.1830
Freundlich	*K*_F_ ((mg g^−1^) (L mg^−1^)1/*n*)	10.7275	8.4217	3.7783
	*n*_F_	1.1192	1.5741	1.3589
	1/*n*	0.8935	0.6353	0.7359
	*R* ^2^	0.8664	0.9615	0.8103
**Temkin**	***K***_**T**_ **(L mg** ^**−1**^ **)**	**3.4470**	**4.7050**	**1.4136**
	***b***_**T**_ **(kJ mol** ^**−1**^ **)**	**172.2090**	**295.0040**	**294.4980**
	***R*** ^**2**^	**0.9767**	**0.9560**	**0.9555**
Dubinin–Radushkevich	*q*_m_ (mg g^−1^)	37.2369	25.9404	24.2617
	*β* (mol^2^ kJ^−2^)	0.2199	0.1096	0.5079
	*R* ^2^	0.9069	0.8622	0.7104
	*E*	4.2650	6.0412	2.8063
Halsey	*N*	−1.1192	−1.5741	−1.3588
	*K*	0.0703	0.0349	0.1643
	*R* ^2^	0.8664	0.9615	0.8103

A value of 0 < *R*_L_ < 1 shows favorable adsorption conditions;

*R*_L_ > 1 shows unfavorable adsorption conditions;

*R*_L_ = 1 shows linear adsorption conditions; and

*R*_L_ = 0 shows irreversible adsorption conditions.

#### 3.8.2 Freundlich's model

Freundlich model is an empirical equation that assumes heterogeneous adsorption due to the diversity of adsorption active sites.^46^ The Freundlich consistent gain from a plot of log *q*_e_*versus* log *C*_e_. The Freundlich linear type isotherm is as demonstrated as follows in eqn (13):13
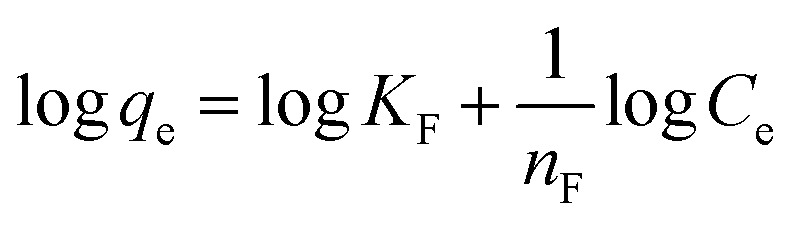
where *K*_F_ ((mg g^−1^) (L mg^−1^)^1/*n*^) is the adsorption capacity, while Freundlich constants characterizes by *n*, separately. Larger value of *K*_F_ point to greater adsorption capacity, while *n*_F_ values designate the preferability of the adsorption process.^53^ The adsorption process is favorable for physical adsorption if *n*_F_ is above unity. The further details of *n*_F_ values can be used to anticipate the adsorption consumption in a calculated adsorption system as shown in Table 2.

**Fig. 9:**
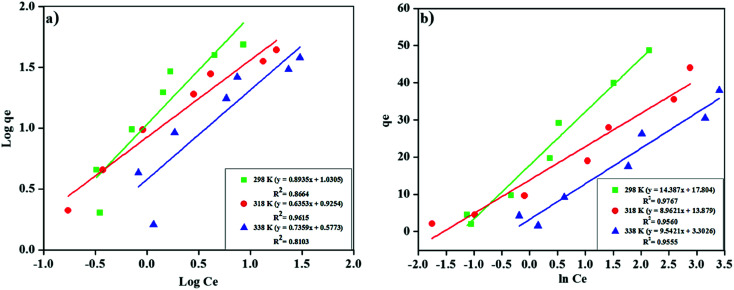
(a) Freundlich's isotherm model and (b) Temkin's isotherm model for the adsorption of RB on MNP-PANI-DICAT at 298 K, 318 K and 338 K.

Freundlich's model result showed (Fig. 9a), is supported with *R*^2^ > 0.81 that the MNP-PANI-DICAT has heterogeneous surfaces as shown in Table 2. The Freundlich constant, *K*_F_ values for the adsorption capacity of RB presented a reduction with the elevated temperature determined as 10.728, 8.421, and 3.778 for 298 K, 318 K, and 338 K correspondingly, which indicated that the adsorption process was exothermic. The *n* values that represent of Freundlich constants were in the range of 2 > *n* > 1 for the adsorption of RB at all the different temperatures, showing that pseudo-linear of the model.

#### 3.8.3 Dubinin–Radusckevich's model

The D–R isotherm study has been generally employed in order to determine the high degree of rectangularity.^48^ The D–R isotherm equation in the linear form displayed below in eqn (14):

14 ln *q*_e_ = ln *q*_m_ − *βε*^2^

where *β* (mol^2^ kJ^−2^) signifies the constant of an adsorption constant acquired from the slope of the linear plot of ln *q*_e_*versus ε*^2^ and *ε*, the Polanyi potential, can be intended by the subsequent equation in eqn (15):

15 *ε* = *RT* ln[1 + 1/*C*_e_]

where *T* is the temperature in Kelvin (K) and *R* is the universal gas constant in kJ (mol K)^−1^. The mean free energy, *E* (kJ mol^−1^) can calculate by the displayed equation in eqn (16):

16 *E* = 2*β*^−0.5^

The designed value of *β* < 1.0 from Dubinin–Radushkevich's isotherm model of MNP-PANI-DICAT for the adsorption of RB represented a rough surface and multilayers structure.

#### 3.8.4 Temkin's model

In addition, other model for the study of indirect adsorbent/adsorbate relations on adsorption is Temkin model.^47^ This isotherm deduced that the heat of adsorption for all the molecules in the layer would linearly decline with coverage and the adsorption is categorized by binding energies in a uniform distribution, catching up to maximum binding energy. The linear form of Temkin isotherm equation eqn (17) is as follows:

17 *q*_e_ = ln *KT* + *β* ln *C*_e_

where *β* = *RT*/*b*_T_. A plot of *q*_e_*versus* ln *C*_e_, constant *K*_T_ and *b*_T_ can be instigate from the intercept and slope. The *b*_T_ (J mol^−1^) is Temkin constant related to the heat of adsorption, while *K*_T_ (L mg^−1^) represents Temkin constant associated to the equilibrium binding energy. Adsorption isotherms of RB on MNP-PANI-DICAT were better fitted by Temkin's model with *R*^2^ > 0.95 for all the calculated temperatures as shown in Fig. 9b. The adsorption was characterized by a uniform distribution of binding energies, up to some maximum binding energy and the heat of adsorption of all the molecules in the layer would decrease linearly with coverage. This shows that MNP-PANI-DICAT has multilayers surface with polymeric polyaniline and imidazolium ring. Therefore, it can predictable that the adsorption system with exist with dissimilar types of interaction such as π–π interaction and hydrogen bonding.

#### 3.8.5 Halsey's model

The Halsey adsorption isotherm is suitable for multilayer adsorption and the fitting of the experimental data to this equation demonstrate the heterogeneous nature of the adsorbent. This model commonly represented as eqn (18):18
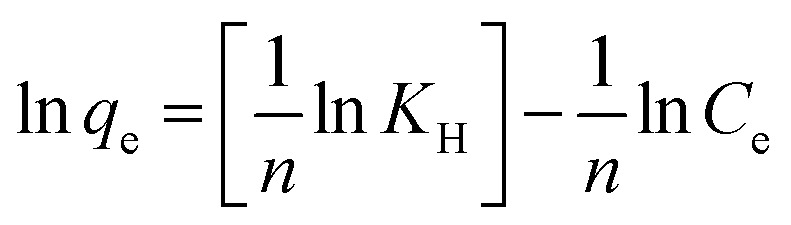
where *K*_H_ and *n* are the Halsey isotherm constant and exponent, respectively. The linear plot of ln *q*_e_*versus* ln *C*_e_ of Halsey's model indicate the heteroporosity (macropore and mesopore) of the adsorbents. Hasley's model also supported which recommended adsorption of RB on the heterogeneous surfaces of MNP-PANI-DICAT.

Table 2 showed the experimental equilibrium data for the adsorption of RB on MNP-PANI-DICAT at different temperatures. In nutshell, the acceptability of the isotherm models to the adsorption behaviour was intermediated *via R*^2^ values. Consequently, the data of adsorption equilibrium was fitted the isotherm models in the order of Temkin > Freundlich > Halsey > Dubinin–Radushkevich's > Langmuir on behalf of the adsorption of RB on MNP-PANI-DICAT.

### 3.9 Adsorption thermodynamics

Gibb's free energy change (Δ*G*°) was calculated *via*eqn (19), while entropy change (Δ*S*°) and enthalpy change (Δ*H*°) were designed from the slope and intercept of the Van't Hoff plot (ln *k*_d_*versus* 1/*T*) using eqn (20) as shown below:^35,54^

19 Δ*G*° = −*RT* ln *k*_d_

20
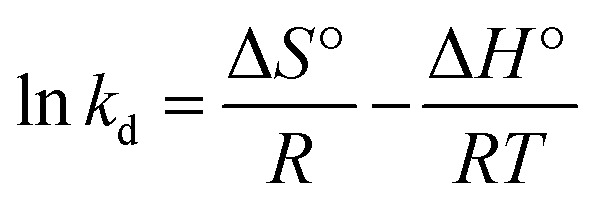
21
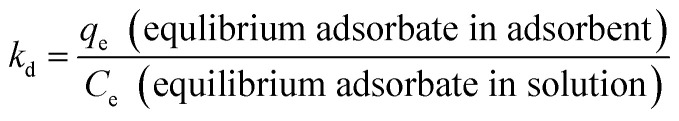
where *R* indicates universal gas constant in kJ (mol K)^−1^, *T* is the temperature (K), while *k*_d_ is the equilibrium constant.^55^

Table 3 reviewed the thermodynamic parameters for the adsorption of RB on MNP-PANI-DICAT showed that the result of Δ*G*° was found to be negative values for the adsorption of RB, which indicated that MNP-PANI-DICAT was undergo process of the thermodynamically feasible and chemically controlled at lower temperature.^35,54^ It also indicated that the adsorption process of RB on the surface of MNP-PANI-DICAT was spontaneous.

**Table 3:** Thermodynamic parameters for RB on MNP-PANI-DICAT

*T* (K)	ln *k*_d_	Enthalpy, Δ*H* (J mol^−1^)	Entropy, Δ*S* (J K^−1^ mol^−1^)	Gibbs energy, Δ*G* (kJ mol^−1^)
298	2.1815	−40.4094	−117.9258	−5404.8362
318	0.9812			−2594.0980
338	0.2589			−727.5547

Upon this, the enthalpy process (Δ*H*°) showed the negative value (−40.41) which indicated that the adsorption process was exothermic. This also be maintained by the diminished of the *K*_F_ values in Freundlich's model, with the decreased of uptake capacity of the sorbent with the increase of the temperature as shown in Table 2. Negative value of Δ*H*° explained that the adsorption process undergoes physisorption and chemisorption of RB on MNP-PANI-DICAT.

Meanwhile, the negative value of entropy, (Δ*S*°) was obtained due to decreasing of randomness at solid-solution interface during adsorption process of RB on MNP-PANI-DICAT.^56^

### 3.10 Analysis of real samples

**Fig. 10:**
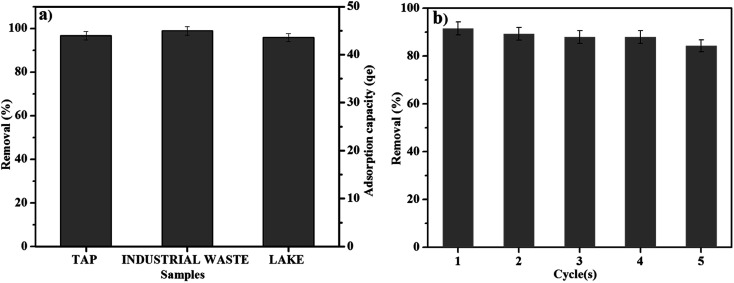
(a) The percentage removal of RB on MNP-PANI-DICAT in real water samples at room temperature and (b) reusability of removal efficiency on MNP-PANI-DICAT adsorbent in five (5) difference run. (Condition: sorbent: 20 mg; initial concentration: 80 mg L^−1^; volume: 10 mL; sample pH at pH 1, and removal time: 60 min, 298 K).

The removal of RB by using MNP-PANI-DICAT in real sample solutions was carried out. The percentage of removal of RB was shown in Fig. 10a for water samples prior to UV-Vis analysis. This spiking analysis using 80 mg L^−1^ of RB solution tested for matrix effect between adsorbent and adsorbate in real water samples. Based on graph, more that 90% of removal of RB indicated that no significant matrix effect in aqueous solutions was observed. Moreover, the adsorption capacity (*q*_e_) for the all real water samples were found to be more than 40 which indicates good binding capacity of MNP-PANI-DICAT (adsorbent) toward RB solution (adsorbate).

### 3.11 Reusability of MNP-PANI-DICAT

Reusability process was beginning after the adsorbent was fully washed with ethyl acetate and then dried 1 hour at 40 °C before the next removal process that slightly modified method from previous articles reported.^57–59^ Based on Fig. 10b, the excellent performance toward removal efficiency was observed which demonstrated good reusability and stability up to 5 cycles of MNP-PANI-DICAT in the removal procedure toward RB sample solution.

### 3.12 Comparison of different sorbents

The developed new material (MNP-PANI-DICAT) was compared with other sorbents and summarized in Table 4. The developed method was exhibits high adsorption capacities (*q*_e_), with lower sorbent dosage and equilibrium time (*q*_*t*_) compared to other previous works. Thus, MNP-PANI-DICAT was considered as a capable sorbent for the removal of RB in aqueous samples.

**Table 4:** Comparison of maximum adsorption capacity of RB on various adsorbents

Sorbent	Sorbent dose (g L^−1^)	*C*_o_ (mg L^−1^)	*q*_e_ (mg g^−1^)	*q*_*t*_ (hours)	Reference
Sodium montmorillonite	0.30	200	42.19	5.00	54
Bagasse pith (BPH)	1.00	600	264.00	5.00	60
Fe_3_O_4_/HA	0.50	50	161.80	0.25	61
Chemically modified solid waste (CMSW)	2.00	50	6.71	1.00	62
**MNP-PANI-DICAT**	**0.20**	**80**	**109.90**	**1.00**	**This work**

## 4 Conclusion

In conclusion, MNP-PANI-DICAT was successfully evaluated as an adsorbent for the adsorption of RB from an aqueous solution. Kinetic analysis showed that pseudo second order equation was best fitted with higher correlation for the adsorption of RB with correlation of determination (*R*^2^) values ranging from 0.9992–1 for the adsorption data with 60 min being the equilibrium time. The isotherm studies were carried out with Langmuir, Freundlich, Temkin, Dubinin–Radushkevich's and Hasley's models to investigate the adsorption behaviour. From the data, Temkin isotherm model was selected as best fitted among other models with *R*^2^ > 0.95 for all studied temperatures for the adsorption of RB on MNP-PANI-DICAT. On the other hand, the negative value of Δ*H*° (−40.4094) indicated that the adsorption process of RB was exothermic. Meanwhile, the negative value of Δ*S*° (−117.9258) was obtained due to decreasing of randomness at solid-solution interface during the adsorption process. The values of Δ*G*° for the adsorption of RB was found to be negative which showed that the process was chemically controlled, thermodynamically feasible, and spontaneous nature of the adsorption processes on MNP-PANI-DICAT.

## Conflicts of interest

The authors have declared that no conflict of interest.

## Supplementary Material

RA-008-C8RA06687F-s001
