# Supplementary material for: Polyaniline modified magnetic nanoparticles coated with dicationic ionic liquid for effective removal of rhodamine B (RB) from aqueous solution
Source: RSC Adv. 2018 Sep 26;8(58):33180–92. doi: 10.1039/c8ra06687f (PMC9086441; doi:10.1039/c8ra06687f)
Supplement: RA-008-C8RA06687F-s001 [file RA-008-C8RA06687F-s001.pdf]

This journal is © The Royal Society of Chemistry 2018

# List of Supporting Information

**Fig. S1** $^{1}H$ NMR interpretation proposed structure of dicationic ionic liquids.

**Fig. S2** TEM analysis 100 nm of (a) native MNP, (b) MNP-PANI, (c) MNPPANI-DICAT and particle diameter distributions (d) native MNP, (e) MNP-PANI, and (f) MNP-PANI-DICAT.

**Fig. S3** Scanning wavelength for effect of time on RB (a) MNP, (b) MNP-PANI and (c) MNP-PANI-DICAT.

**Table S1** Main IR frequency with assignments and $^{1}H$ NMR chemical shift (δ) of dicationic ionic liquid**.**

**Table S2** Weight loss profiles for TGA analysis of MNPs, MNP-PANI and MNP-PANI-DICAT.

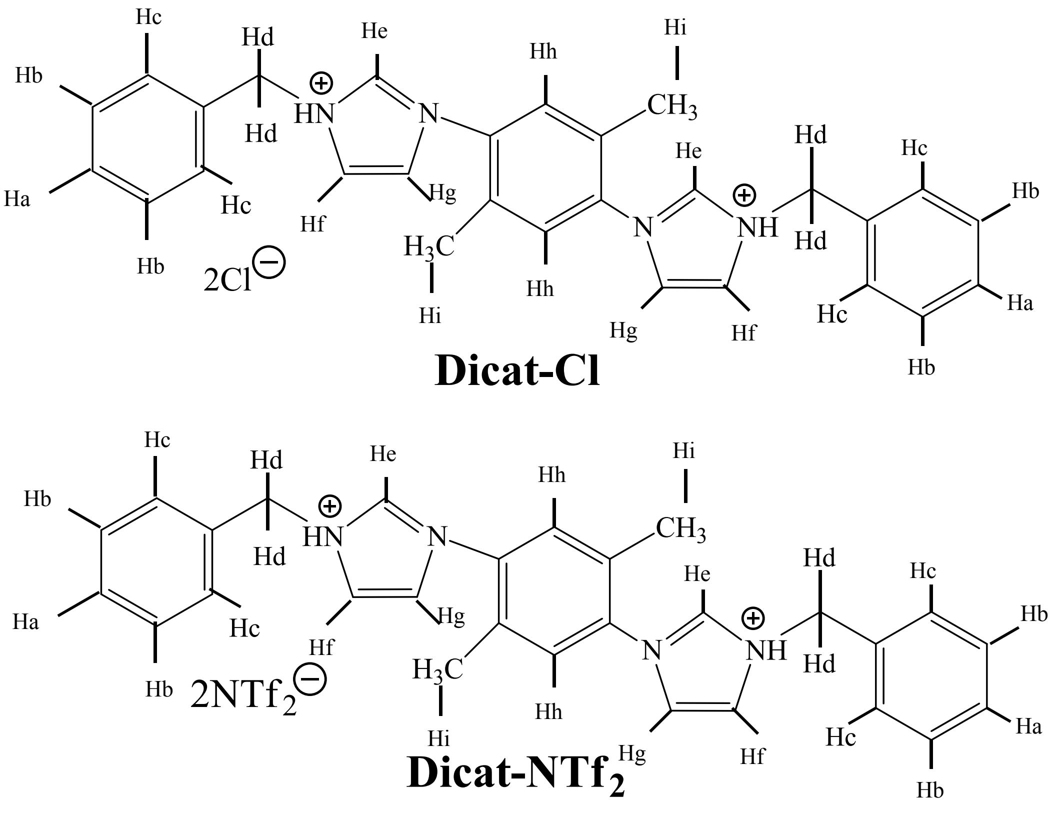


**Fig. S1** $^{1}H$ NMR interpretation proposed structure of dicationic ionic liquids.

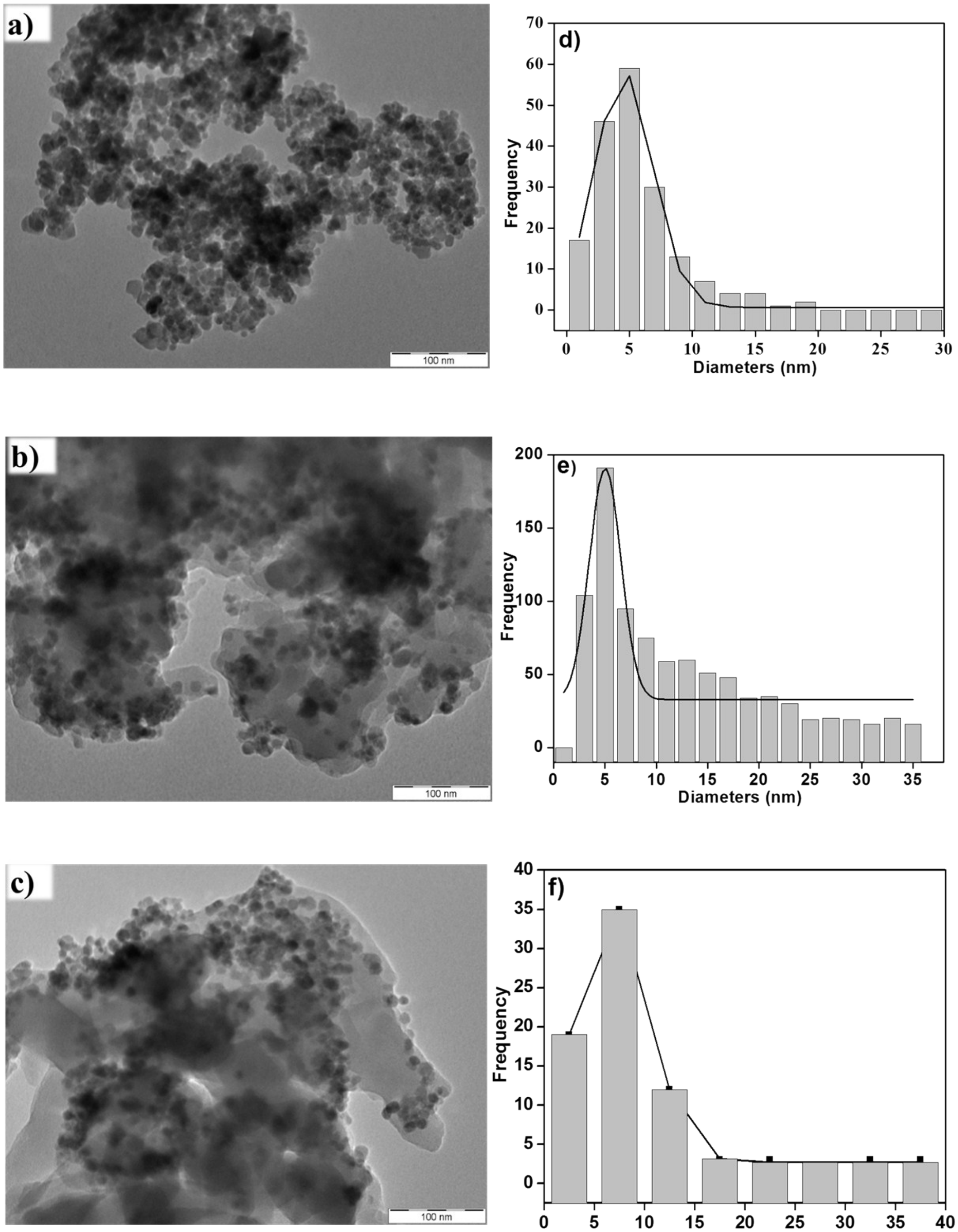


**Fig. S2** TEM analysis 100 nm of (a) native MNP, (b) MNP-PANI, (c) MNP-PANI-DICAT and particle diameter distributions (d) native MNP, (e) MNP-PANI, and (f) MNP-PANI-DICAT.

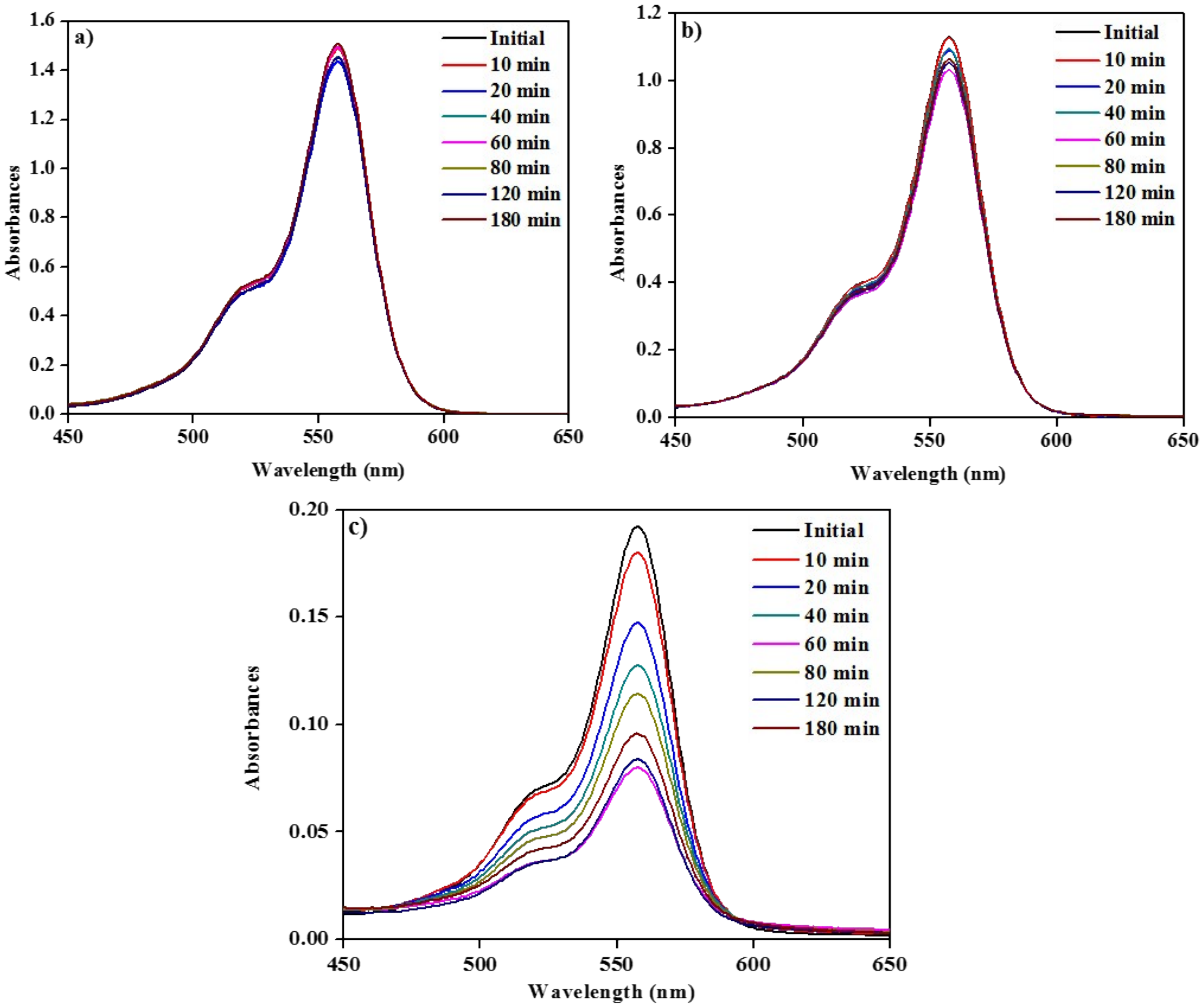


**Fig. S3** Scanning wavelength for effect of time on RB (a) MNP, (b) MNP-PANI and (c) MNP-PANI-DICAT.

**Table S1** Main IR frequency with assignments and $^{1}H$ NMR chemical shift (δ) of dicationic ionic liquid.

| **Assignments:** | **DICAT-Cl** | **DICAT-NTf$_2$** |
|---|---|---|
| | Wavenumber ($cm^{-1}$): | |
| N-H stretching | 3444.71 | 3627.21 |
| C=C aromatic ring | 1647.15 | 1634.09 |
| $-CH_2$ | 1458.54 | 1455.98 |
| $-CH_3$ | 1353.42 | 1353.62 |
| C-C aromatic ring | 1506.50 | 1515.93 |
| C-N aromatic ring | 1233.43 | 1201.31 |
| -Cl | 715.64 | - |
| **Proton (Changes):** | Chemical shift (δ) | |
| Ha (+0.019) | 7.077 | 7.096 |
| Hb (+0.079) | 7.111 | 7.190 |
| Hc (+0.025) | 7.061 | 7.086 |
| Hd (+0.031) | 5.030 | 5.061 |
| He (+0.194) | 7.461 | 7.655 |
| Hf (-0.007) | 7.006 | 6.999 |
| Hg (+0.031) | 7.080 | 7.111 |
| Hh (+0.030) | 6.819 | 6.849 |
| Hi (+0.010) | 2.217 | 2.227 |

**Table S2** TGA analysis of MNPs, MNP-PANI and MNP-PANI-DICAT

| Sample | Region (°C) | Weight loss (%) | Assignment |
|---|---|---|---|
| **MNPs** | (35 – 129) | 2.3 | Water loss/ moisture |
| **MNP-PANI** | (42 – 126)<br>(362 – 688) | 7.9<br>67.8 | Water loss/ moisture<br>Aniline polymer |
| **MNP-PANI-DICAT** | (43 – 102)<br>(364 – 739) | 6.8<br>66.6 | Water loss/ moisture<br>Imidazolium, Aniline polymer |
